# Advance care planning in older hospitalised patients following an emergency admission: A mixed methods study

**DOI:** 10.1371/journal.pone.0247874

**Published:** 2021-03-05

**Authors:** Anna-Maria Bielinska, Stephanie Archer, Adetokunbo Obanobi, Gehan Soosipillai, Lord Ara Darzi, Julia Riley, Catherine Urch

**Affiliations:** 1 Department of Surgery and Cancer, Imperial College, London, United Kingdom; 2 Imperial College Healthcare NHS Trust, London, United Kingdom; 3 The University of Cambridge, Cambridge, United Kingdom; 4 The Royal Marsden NHS Foundation Trust, London, United Kingdom; University of Mississippi Medical Center, UNITED STATES

## Abstract

**Introduction:**

Although advance care planning may be beneficial for older adults in the last year of life, its relevance following an emergency hospitalisation requires further investigation. This study quantifies the one-year mortality outcomes of all emergency admissions for patients aged 70+ years and explores patient views on the value of advance care planning following acute hospitalisation.

**Method:**

This mixed methods study used a two-stage approach: firstly, a quantitative longitudinal cohort study exploring the one-year mortality of patients aged 70+ admitted as an emergency to a large multi-centre hospital cohort; secondly, a qualitative semi-structured interview study gathering information on patient views of advance care planning.

**Results:**

There were 14,260 emergency admissions for 70+-year olds over a 12-month period. One-year mortality for admissions across all conditions was 22.6%. The majority of these deaths (59.3%) were within 3 months of admission. Binary logistic regression analysis indicated higher one-year mortality with increasing age and male sex. Interviews with 20 patients resulted in one superordinate theme, “*Planning for health and wellbeing in the spectrum of illness”*. Sub-themes entitled (1) *Advance care planning benefitting healthcare for physical and psycho-social health*, (2) *Contemplation of physical deterioration death and dying* and 3) *Collaborating with healthcare professionals to undertake advance care planning*, suggest that views of advance care planning are shaped by experiences of acute hospitalisation.

**Conclusion:**

Since approximately 1 in 5 patients aged 70+ admitted to hospital as an emergency are in the last year of life, acute hospitalisation can act as a trigger for tailored ACP. Older hospitalised patients believe that advance care planning can benefit physical and psychosocial health and that discussions should consider a spectrum of possibilities, from future health to the potential of chronic illness, disability and death. In this context, patients may look for expertise from healthcare professionals for planning their future care.

## Introduction

In order to support patient autonomy, healthcare professionals need to provide patients with an opportunity to discuss their preferences for care, understand their disease trajectory and treatment options [1, 2]. In particular, there is an ethical duty to explore patients’ wishes in order to make informed choices, particularly towards the end of life [3, 4]. Advance care planning (ACP) provides a mechanism for such conversations to take place [5].

ACP covers a very broad spectrum of care planning, and includes future care planning, urgent care planning, and end-of-life planning. Future care planning offers patient-centred planning, focussing on understanding well-being, health and illness for persons with several years’ prognosis [6]. Urgent care planning is mostly aimed at persons with a limited, 1–2 years prognosis, focussing on medical options for care, including treatment limitations, understanding illness trajectory and may include cardio-pulmonary resuscitation decisions [7]. Urgent care plans can be shared with the out-of-hours healthcare teams to guide care during an emergency in accordance with the patients’ wishes, such as Coordinate My Care -a digital urgent care planning service [7]. End-of-life care plans, aimed at persons with a prognosis of 12 months or less, focus on providing patient-centred terminal care, potentially discussing end-of-life care wishes, place of care and preferred place of death [8].

When a patient is approaching the end of their life—a likelihood of dying within one year—healthcare professionals are encouraged to engage with ACP by discussing care preferences with patients regarding choices for care [9]. Since the majority of deaths are in older adults [10], improving ACP in older age is key for providing high quality care. Attention has been drawn to the relevance of ACP in hospitals [11, 12], particularly the need to improve the uptake of ACP in older, frail accessing acute medical care [13]. Cohort studies in Scotland have looked at one-year mortality outcomes in hospitals -one demonstrating that 28.8% of inpatients died within 12 months [14] and another reporting a 22.4% death rate following an emergency medical admission [15]. The Scottish one-year mortality cohort studies were mostly in older patients—64.1% were aged 65 years+ in one cohort [14] and 62.6% were aged 60+ in a more recent study [15]. However, no comparable studies to date have looked at an English sample.

Among older people who have had emergency admissions there are some who are in the end-of-life phase, for whom ACP is crucial. The prevalence of ACP in the acute hospital environment is addressed in research elsewhere [13, 16], and indicates that uptake of ACP is low, including in older and seriously ill inpatients [16]. We hypothesise that older hospitalised patients may benefit from ACP and that an emergency admission may be a suitable catalyst for starting ACP. Furthermore, there is a lack of qualitative research investigating the views towards ACP of older adults who have been hospitalised following an emergency. Therefore, the aim of this study is to employ a two-stage mixed methods approach to (a) identify the one-year mortality of patients aged 70 and over admitted as an emergency and (b) gather information on experiences and views towards ACP within this group.

## Methodology

### Design overview

In order to achieve our aims, we utilised a mixed methods design, including a quantitative analysis of data from emergency admissions from 2014–2015, with one-year mortality figures calculated for 2015–2016 and qualitative data collected from interviews in 2017 on views of advance care planning (ACP). The interview participants were recruited in 2017, one year after the mortality outcomes study, since the quantitative study informed the qualitative component of the research.

#### Patient and public involvement

In addition to the literature regarding gaps in knowledge on ACP in later life (including [5, 6, 8, 17]), input from palliative care clinicians and a health psychologist, the semi-structured interview schedule was co-designed and pilot tested with a sample of patients and carers who had a specific interest in advance care planning [18]. This patient and carer interest group, who had been convened to contribute to the design and development of NHS advance care planning services (Coordinate My Care), gave feedback on the overall study design and shaped the semi-structured interview topic guide [18].

#### Ethics

The quantitative study was performed as part of service evaluation as per HRA guidance. Research ethics approval for the qualitative study was granted by the Hampstead Research Ethics Committee (REC reference number 16/LO/1798) and the Health Research Authority (IRAS project ID 208736). All participants who took part in interviews gave written informed consent to participate.

*Setting*. The study was conducted at Imperial College Healthcare NHS Trust, a large London multi-centre hospital trust, providing acute and specialist health services for a diverse socio-economic urban population.

### Quantitative study

#### Participants and recruitment

Data from a 12-month cohort of emergency admissions (May 2014-April 2015) were captured and tracked longitudinally for a further year to establish one-year mortality. At the time of index admission, participants were aged 70 years or over, and had undergone emergency admission to the hospital trust, with a minimum one overnight stay. This study used an age-criterium of 70+ years for older patients. Although there is no universal chronological definition for later life, there are studies using 70 as an age cut-off in both malignant [19] and non-malignant [20] disease management. There are also self-care and healthy ageing strategies aimed at the over 70s in England [21]. Furthermore, the UK Office for National Statistics have considered “age 70 the new age 65” [22], suggesting 70 as an appropriate cut off for older age.

#### Measures—data source and extraction

Data were gathered from the Cerner electronic data warehouse, which provides a real-time, up-to-date digital medical record for patients at the hospital Trust [23]. Data related to admissions rather than to individual patients.

#### Statistical analysis

Data analysis was performed using SPSS Windows (Version 23.0). Initially, admission data were analysed for both sexes and for all ages for 70-year-olds and above. Thereafter, data were stratified according to sex and three age bands: 70–79 years, 80–89 years and 90 years and over. Odds ratios and Chi-squared statistics were calculated for the influence of gender on mortality. Survival times were analysed using the Kaplan-Meier procedure and compared using the log-rank test. Binary logistic regression was performed to assess the effect of the variables of sex and age on one-year mortality.

### Qualitative study

#### Participants and recruitment

Participants were patients aged 70 years and over who had been admitted for at least one overnight stay in hospital following an emergency admission regardless of diagnosis. The patient’s admitting team assessed their eligibility, whether they were sufficiently clinically stable to give an in-depth interview, able to speak English and give valid informed consent. Patients were not eligible to participate in this study if they lacked capacity, had a terminal diagnosis or were admitted to a high dependency or intensive care unit and therefore clinically unable to participate. Potential participants were recruited consecutively according to the order of a random computer-generated list and were approved by ward managers as being sufficiently stable to be invited for an interview.

#### Measures -semi-structured interviews

Face-to-face semi-structured interviews were conducted in hospital with each participant by AMB, a doctor outside the patient’s clinical care team, with a research interest in ACP. The interviews were based on a semi-structured interview topic guide. The interview explored the preferred language of future care planning, preferred content (modified from Boyd [6]), perceived impact in terms of advantages and disadvantages and attitudes to sharing future care plans. Participants were also shown an information leaflet from an ACP service (Coordinate My Care), of a patient who had benefitted from urgent care planning, to trigger reflection on Electronic Palliative Care Coordination Systems. Data collection continued until data saturation was reached.

#### Thematic analysis

Interviews were digitally audio-recorded and transcribed verbatim, using pseudonyms. Transcriptions were checked for accuracy alongside recordings. All transcripts were thematically analysed [24], with formal coding manually line-by-line and notes written to support data patterns (by AMB). A random sample of n = 2 transcripts were second-coded by another researcher (GS), revealing high concurrence. The sub-themes, themes and thematic map generated by AMB were reviewed with SA and CEU, with any variances reviewed and consensus established through discussion. Quotations from participants were used to demonstrate themes and sub-themes.

## Results

### Quantitative results

There were 14,260 emergency admissions aged 70+years over the study year. Incomplete records for the date of death were excluded (n = 21; 0.15% of admissions).

Within the cohort of admissions, 50.6% were female and 49.4% male. In total, 48.1% were aged 70–79 years, 39% were 80–89 years and 12.9% were 90+ years.

One-year combined mortality for male and female admissions to all specialties aged 70 years and over was 22.6%. The majority of deaths (59.3%) were within 3 months of admission; 18.1% died between 3–6 months, 12.4% died between 6–9 months and 9.7% died between 9–12 months.

**Fig 1:**
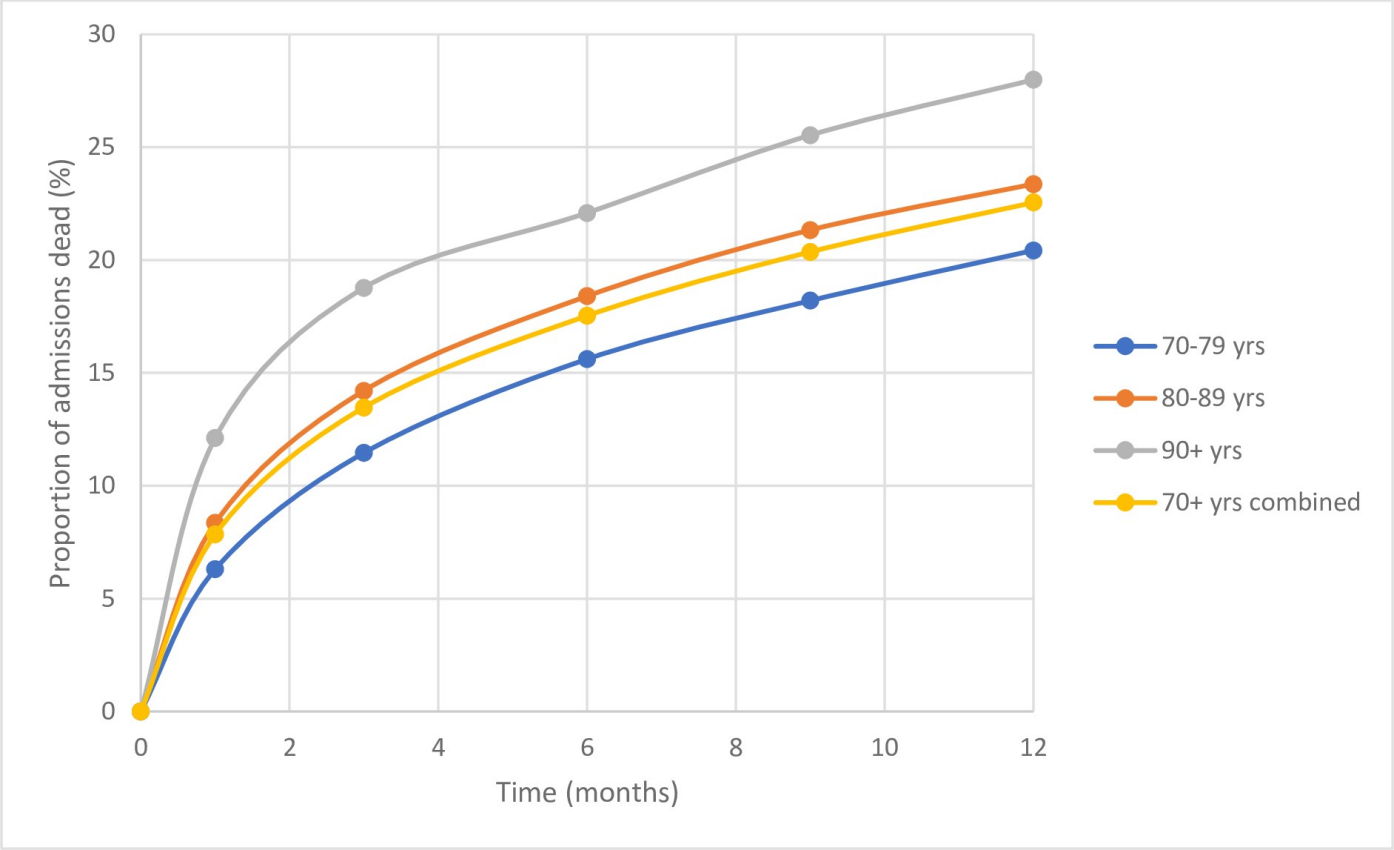
Mortality over time for emergency male and female admissions for 12-month cohort.

One-year mortality for both sexes combined rose with age band from 20.43% for 70–79 year-olds, to 23.37% in 80–89 year-olds and to 27.99% in 90+ year-olds (Fig 1).

Kaplan Meier survival analysis showed a significantly reduced survival with increasing age. The longest survival was in the 70–79 year-old category, with an average mean survival of 312.5 days (log rank test, P < 0.001), followed by 80–89 year olds and 90+ year olds. The median survival was not reached. Binary logistic regression analysis for age indicates higher one-year mortality with increasing age (OR 1.026; 95% CI 1.021–1.032; P < 0.001). For every 1-year increase in age there is a 2.6% increase in one-year mortality.

Men had a higher one-year mortality (25.02%) compared to women (20.13%) across all age bands.

Kaplan Meier survival analysis showed a reduced survival for male compared to female emergency admissions (log rank test, P < 0.001). The median survival was not reached. Binary logistic regressions analysis indicated higher one-year mortality for males (OR:1.40; 95% CI < 1, P < 0.001). The odds ratio for death for 1-year mortality for males compared with females is 1.32, adjusted for age, indicating higher mortality for male admissions (P < 0.001).

### Qualitative results

One superordinate theme, *Planning for health and wellbeing in the spectrum of illness*: *“well I suppose that’s what it’s all about…health and medicine isn’t it*?*”*, from a larger body of work is reported here. Overall, participants perceived ACP within the context of their experiences as a patient, at different junctures in their life and their overall experience of healthcare. Some older adults felt that ACP best suited a medical paradigm. There was evidence of older individuals using their experience of a medical emergency, chronic disease or serious illness to consider ACP. The analysis focused around three sub-themes entitled (1*) Advance care planning benefitting healthcare for physical and psycho-social health*, *(2) Contemplation of physical deterioration death and dying*, (3) *Collaborating with healthcare professionals to undertake advance care planning*. All quotations have been anonymised, using either pseudonym surnames or single initials chosen by participants at the time of interview.

#### Sub-theme 1: ACP benefitting healthcare for physical and psycho-social health

Participants reported that ACP related to both physical and psycho-social health. Participants identified a role for ACP in looking after physical welfare including monitoring, safety and care in chronic illness and when necessary, to help in a crisis. Miss JG, for example, when asked about what ACP means to her, described regular community checks at home from health and social care on her father who had chronic renal failure:

“They came in for my father…as he had kidney failure…and erm, we avoided having him on… dialysis because he had to go a long way on patient travel and that can take hours…and some of the doctors were telling me it can make him quite ill…so he wanted to be at home…so carers came in… …and apart from that nurses came into check him every day….”

Some older persons believed that longevity of older adults should be supported by medical professionals and that this could be linked to a future care planning discussion. Mrs WP stated that ACP might provide additional attention which might improve the physical outcome of a person who has deteriorated:

“Yeah, do things earlier. And who knows, the patient might, because they’re getting a little bit of extra help, or not extra help, but the help that they need and the attention and stuff …they might improve”.

Others had a more negative view of what care planning is in terms of physical benefits, thinking it is restricted to personal care, hygiene and regimented routine. Mrs Rides explained:

“I really thought care plan smacks of somebody washing you and dressing you and you need to be taken to a club or something once a week just to get out the house……

Participants also expressed a psycho-social dimension to ACP, including emotional well-being, particularly maintaining hope, gratitude and feeling at peace with decisions, sometimes linked to spirituality. There was also a need to be aware of vulnerability and maintaining resilience in planning future care. Mrs Blue stated that use of a gentle conversational style in ACP discussions is important psychologically:

“don’t bring that “Oh my God!” in, do you know what I mean? Oh God what am I going to do now? …If you can do it in a sort of round-about way -what do you think about this, what do you think about this….”

Mrs WP linked discussing prognosis in ACP with maintaining psychological well-being, enabling families to make plans so that there is peace of mind at the end of life:

“Well, I think everybody would want to know their prognosis and I think the doctors should tell them…they can get in touch with their family and tell them … so they can all get together …they’ve only got six months to live, they can sort their plans out….so when they pop off, they think, “I’ve done everything, I’ve looked after everybody…now I can go with ease”.

However, others believed that avoiding contemplating the future in ACP could be protective. Mrs Krohn described how she wanted to avoid facing the future:

“And I haven’t really thought about what I want to do in the future because not all that much in the future for me.”

Some participants felt that psycho-social aspects of ACP could be heavily influenced by the personalities of care providers. Mr J stated that the workability of “Coordinate My Care” relied on a therapeutic relationship and receiving empathy:

“No, I think the most important thing probably is going to be the relationship that you can establish with the key individuals really…So, you’ve got to have a degree of empathy…if you leave the personalities out of it, it doesn’t always work right”.

Overall, participants believed that ACP could have a holistic role in benefiting their physical and psycho-social well-being in later life. Participants expressed a variety of ways that ACP discussions facilitated by family and healthcare professionals that might support this.

#### Sub-theme 2: Contemplation of physical deterioration, death and dying

Participants linked ACP with a need to contemplate physical deterioration. This generally concentrated on disease and ageing, one’s own personal mortality and experiences of chronic illness, disability and death. For example, Mrs B- considered the impact on quality of life due to physical deterioration and severe disability when reflecting on ACP:

“You came into this world with a healthy body and then all of a sudden you have a stroke and you’re like a vegetable–no, that wouldn’t be for me, definitely not.”

Participants positioned terminal illness as part of the spectrum of disease and described that accepting a limiting prognosis was an important part of ACP. For example, Mrs M stated that discussing prognosis was important if it was likely that physical deterioration would be progressive:

“you should always discuss it…because if you’re not going to get any better you know what is coming…innit (sic)?”

At times, participants acknowledged the potential futility of treatment as part of contemplating death and realising the potential burden associated with illness and treatment at the end of life. When discussing her views on ACP, Mrs Ride recounts and values previous candid discussions with an intensive care doctor regarding her late husband, who was terminally ill, and the medical futility of critical care interventions:

“I’m sure he wouldn’t want his life prolonged more any longer because there was no hope, and we agreed to let him fade away and I feel I did the right thing…”

Participants also discussed the value of preparedness for care arrangements following illness and for terminal care. Mrs ZZO explained that her personal interpretation of ACP involved having discussions to feel prepared for end-of-life care:

“Well, there’s a certain amount of it includes care planning when someone becomes ill and becomes unable to cope with things…and other than that there’s the care when someone is dying, and you’ve got to prepare them for it and that I think is quite important”.

Participants acknowledged that for some older persons, practical preparation for death itself, namely with funerals, burial arrangements and on occasion organ donation choices were crucial. Mr N discussed his wish for cremation and directly linked this to ACP:

“I told them that I expect when I shuffle off this mortal coil to be cremated.”

In summary, ACP triggered participants to contemplate potential deterioration in old age and to reflect on their own mortality. Participants believed this might help with feeling prepared for the end-of-life, with an emphasis on decision-making in end-of-life care.

#### Sub-theme 3: Collaborating with healthcare professionals to undertake advance care planning

Participants recognised the expertise and leadership of professionals and the role this may have in ACP. Some participants felt that the need for input from patients may be limited and some were more open to be led by others. Regarding planning future care, Mrs B stated:

“Yeah, but I’d rather have a doctor explain to me what the situation was, and I’d go along with them, you know what I mean?”

Mr Glass recommended strong leadership from medics in ACP, stating there are flaws in involving the government and considers medical input best:

“…and I got a feeling it might be best left to the experts, such as medical experts… in this respect…”

Further to this, a one-to-one discussion with a clinician was valued in ACP. When asked if he would discuss ACP with a loved one, Mr C explained that he is socially isolated and is more in favour of a discussion with a medical professional:

“I would assume that it would be a one-to-one discussion with the medical personnel and the patient involved”

Participants held varying views on who was best placed to have ACP discussions. Some placed emphasis on the importance of GP led discussions, whereas others felt that hospitals were better informed. The role of the GP’s insight in opening discussions was highlighted by Mr H who described that although he does not have a care plan with the GP, they have tried to probe him on his readiness to have a care plan:

“Like he asks me some amazing questions on the future and what I think. You know? Shows if you really, you are into it.”

Mr C agreed that accident and emergency doctors should be the ones who are privy to information within ACP, and explained their unique situation in influencing outcomes in an acute situation:

“I would imagine because he or she is the nearest in time and space and to the situation”.

Some participants especially valued interdisciplinary teamwork of professionals in ACP. This often included the integration of planning across primary and hospital sectors, together with the support of family. For some, discharge from hospital was the optimum time for ACP to happen, as described by Mrs WP:

“You know, when a person’s in hospital and she’s had the treatment and she’s better and they say, “Look, you’re going home but we would like to put this into progress,” you know, and bring the family up, just have a nice little family discussion and then set it in motion. And then they can get the GP to keep an eye on her once he’s furnished with all the information.”

Overall, participants felt that successful ACP relied on specialist support from healthcare professionals, including as part of a consultation in primary or hospital care, or as part of discharge planning from hospital to the community.

## Discussion

This is the first longitudinal cohort study in England to assess one-year mortality in the over 70s population following emergency hospitalisation, irrespective of diagnosis, across both medicine and surgery. The data indicate that the one-year mortality for emergency admissions for patients aged 70+ is approximately 20%, with most deaths occurring within 3 months of admission. As such, our study shows that a large number of older patients admitted as an emergency are in the last year of life. The qualitative data demonstrate older patients’ potential openness to discuss a range of possibilities for the future, including reflecting on their mortality and potential vulnerability, as well as ACP playing a role in enhancing their physical and psycho-social health. The patients highlighted that expertise and leadership from professionals has a role in supporting ACP in this context. This analysis suggests that healthcare professionals should consider discussing ACP with older adults who are hospitalised after an emergency, noting the potential role in both supporting patients’ mental and physical well-being, and where appropriate, preparedness for deterioration and death, particularly for those with limited prognoses.

### Comparison with existing literature

Our study identified one-year mortality in the over 70s following emergency admission at 22.6%, which is higher than the overall mortality figures for adults aged between 70-89-years-old for deaths registered in England and Wales (from 2.79% for females aged 70–79 years, to 12.9% males aged 80–89 years) [25]. This may be due to the burden of co-morbidity in those admitted as an emergency or the nature of the presenting emergency [26]. Our study confirms the literature which highlights that a large proportion of hospital inpatients may be in the last year of life [14], particularly following an emergency admission [15]. Acute hospital data from New Zealand shows 19.8% of inpatients fulfilled at least one of the Gold Standards Framework prognostic criteria, suggesting they were likely to be in the last year of life [27]. Our data indicate that one-year mortality was higher in males and increasing with age band, supporting Scottish studies [14, 15].

In this study, patients reported that ACP could benefit physical and psycho-social health. Indeed, a number of studies describe ACP as part of chronic disease management [28], although the literature focuses on improved concordance of care, particularly at the end of life, as opposed to improved disease outcomes [29]. There is also evidence in the literature that ACP can improve peace and mental wellbeing, although this is not universal [30, 31], and may differ between different racial or cultural groups [32].

The data in our study indicate that contemplating physical deterioration, death and dying is an important component of ACP. This is supported by a systematic review of ACP with hospital inpatients who experience significant clinical deterioration, which found that earlier engagement in ACP could improve person-centred care and referral to specialist palliative care [33]. Some studies demonstrate that inpatients wish to discuss potential future health outcomes through ACP, although some inpatients report that ACP reduced hope of better times in the future [34]. Furthermore, the qualitative analysis presented here indicates that expertise and leadership from professionals underpins successful ACP, supporting research elsewhere suggesting that poor communication or knowledge from physicians were barriers to ACP [34] and that discussions with trained healthcare professionals were crucial to make informed decisions [35].

### Clinical implications

Most doctors recognise their duty to begin ACP discussions in frail and older adults, but absence of a precipitating event limits this [17]. Our data suggest an emergency admission to hospital for a patient aged 70+ years is a valuable opportunity to engage patients in ACP. Clinicians may have a duty of care to consider discussing ACP as part of shared decision making on a patient-by-patient basis following acute hospitalisation among older adults. This may be particularly important as the results of this study show that approximately one fifth are in the end-of-life phase and that older patients seek to collaborate with clinicians in planning their future care. The need for shared decision making is highlighted by research elsewhere [17] and shows that a low proportion of frail older adults have advance care plans, despite being potentially open to discussions, prompting the question “Do the elderly have a voice?”. This is concordant with studies suggesting that the uncertainty surrounding acute illness and hospitalisation may be a trigger for some patients to discuss ACP, although this is not universal [34].

As ACP is considered to be an umbrella term for a spectrum of discussions with individuals at different stages of illness and differing prognoses, an emergency admission in the over 70s can therefore aid recognition of the last year(s) of life and trigger tailored ACP. We propose that for the 80% of emergency admissions with a prognosis exceeding one year, a general future care planning discussion should be initiated to discuss future care preferences. The majority of patients can use this as opportunity to discuss future health and well-being. For the 20% of admissions with a prognosis of less than one year, we suggest urgent care planning, using clinical judgement, focussing on coordinating care services and understanding preferred place of care and death. For the proportion of patients with a prognosis of under 3 months within this subset (14% of emergency admissions), we propose end-of-life care planning.

Performing frailty screening in the emergency department by geriatricians could help target ACP appropriately for those with a prognosis of under 1 year and there is an increasing recognition of the need to address ACP in hospitalised, frail older adults [36]. The Gold Standards Framework Proactive Identification Guidance (GSF-PIG) are used to identify those who may be in the last year of life in hospitals to target ACP appropriately, using the “Surprise Question” (“Would you be surprised if the patient were to die in next year, months, weeks, days?”) together with other indicators of deterioration [37]. The Supportive and Palliative Care Indicators Tool (SPICT) is used to help identify older persons with deteriorating health with palliative care needs [38]. Other risk prediction tools validated for older people in the emergency department to predict the risk of death within a year might be suitable for targeting ACP in this setting -the ISAR tool [39], The Simple Clinical Score [40], the HOMR index for 1-year [41] and 6 month mortality [42] and the CriSTAL score for death within 3 months [43].

The study underpins the need to share electronic, patient-accessible care plans across multiple sites and out-of-hours care providers to improve communication in ACP, including in relation to acute hospitalisation. In Scotland, chronic disease anticipatory planning is supported using the NHS Key Information Summary Plan [44]. In English GP practices, NHS Direct Enhanced Services strategized to avoid unplanned admissions pro-actively with a personalised care plan for the 2% most vulnerable registered patients [45]. However, normalisation of ACP has not occurred [16], and unscheduled admissions remain high [10]. Currently, the medical model is reactive care [10]; however, ACP with digital sharing of plans represents a disruptive technology, thereby reversing the normal model. To normalise ACP, health policies must recognise the work and time required, incorporating it into the contract of clinicians to do the work up-front, in a ‘spend to save’ type of program.

### Strengths and limitations

A key strength of the quantitative component of the study is that the electronic medical record system (Cerner), uses record linkage of hospital data from one of the largest NHS Trusts in England with national death registration records, for tracking mortality outcomes. Another advantage of the quantitative data that it is the first study to assess one-year mortality in the over 70s population following emergency admission, encompassing all medical and surgical admissions, irrespective of diagnosis, including long stays over 21 days or complex inpatient geriatric admissions. Other studies exclude geriatric long stays [14] or emergency surgical admissions [15]. The main benefit of the interview component is the investigation of the views of older adults admitted to hospital following an emergency, who have direct experience of hospitalisation, emergency care and may therefore benefit from improvements in ACP in this setting. A diverse range of views were captured, since patients were admitted to both medical and surgical specialties and not excluded on the basis of any diagnosis. The study was further strengthened by involving patients and carers in the co-design of the semi-structured interview topic-guide and linguistic approach [18].

The multiphase project investigating ACP in later life was conducted over several years involving several stakeholders, including older patients, carers, clinicians, with the quantitative component informing the qualitative component. However, the results hold relevance for older patients and clinicians, since approaches to discussing ACP are ever pertinent [46], particularly in the under-researched frail and hospitalised population [36].

A limitation of our quantitative component of the study is that it used an incident sample relating to admissions, rather than individual patients. Therefore, our data are affected by patients with multiple admissions. However, surrogate patient level analysis was obtained via first admission data, showing comparable one-year mortality with patient-level data (18.9%) and admission-level data (22.6%).

For the qualitative component, the time-pressured environment may have restricted the depth of the interviews. It is a limitation that older patients who were most acutely unwell, including those with life-threatening emergencies on high dependency units or intensive care units were not interviewed. These patients may have different perspectives on ACP based on their experience in a critical care environment. A further consideration is that the study under-represents the views and experiences of patients with dementia, since participants needed to have capacity in order to consent to the study.

## Conclusion

Since the one-year mortality of patients aged 70+ years admitted as an emergency is approximately 1 in 5, an emergency hospitalisation in this cohort can aid recognition of the last year(s) of life and trigger tailored ACP. Older hospitalised patients believe that ACP is worthy of consideration, taking into account individual circumstances and experiences. An emergency hospital admission should serve as a catalyst to discuss a spectrum of all relevant issues through ACP from the potential of chronic illness, disability and death, to future health and well-being. Expertise and leadership from professionals have a role in supporting ACP in this context. An emergency admission to hospital for an older adult may therefore provide an opportunity to ask meaningful questions and discuss future healthcare preferences.
